# What do evidence-based secondary journals tell us about the publication of clinically important articles in primary healthcare journals?

**DOI:** 10.1186/1741-7015-2-33

**Published:** 2004-09-06

**Authors:** Kathleen Ann McKibbon, Nancy L Wilczynski, Robert Brian Haynes

**Affiliations:** 1 Health Information Research Unit, Department of Clinical Epidemiology and Biostatistics, McMaster University Faculty of Health Sciences, Room 3H6 Health Sciences Center, 1200 Main Street West, Hamilton, Ontario, Canada. L8N 3Z5; 2 Center for Biomedical Informatics, School of Medicine, 8084 Forbes Tower, 200 Lothrop Street, University of Pittsburgh, Pittsburgh, PA, USA. 15213-2582; 3 Department of Medicine, McMaster University Faculty of Health Sciences, Health Sciences Center, 1200 Main Street West, Hamilton, Ontario, Canada. L8N 3Z5

## Abstract

**Background:**

We conducted this analysis to determine i) which journals publish high-quality, clinically relevant studies in internal medicine, general/family practice, general practice nursing, and mental health; and ii) the proportion of clinically relevant articles in each journal.

**Methods:**

We performed an analytic survey of a hand search of 170 general medicine, general healthcare, and specialty journals for 2000. Research staff assessed individual articles by using explicit criteria for scientific merit for healthcare application. Practitioners assessed the clinical importance of these articles. Outcome measures were the number of high-quality, clinically relevant studies published in the 170 journal titles and how many of these were published in each of four discipline-specific, secondary "evidence-based" journals (*ACP Journal Club *for internal medicine and its subspecialties; *Evidence-Based Medicine *for general/family practice; *Evidence-Based Nursing *for general practice nursing; and *Evidence-Based Mental Health *for all aspects of mental health). Original studies and review articles were classified for purpose: therapy and prevention, screening and diagnosis, prognosis, etiology and harm, economics and cost, clinical prediction guides, and qualitative studies.

**Results:**

We evaluated 60,352 articles from 170 journal titles. The pass criteria of high-quality methods and clinically relevant material were met by 3059 original articles and 1073 review articles. For *ACP Journal Club *(internal medicine), four titles supplied 56.5% of the articles and 27 titles supplied the other 43.5%. For *Evidence-Based Medicine *(general/family practice), five titles supplied 50.7% of the articles and 40 titles supplied the remaining 49.3%. For *Evidence-Based Nursing *(general practice nursing), seven titles supplied 51.0% of the articles and 34 additional titles supplied 49.0%. For *Evidence-Based Mental Health *(mental health), nine titles supplied 53.2% of the articles and 34 additional titles supplied 46.8%. For the disciplines of internal medicine, general/family practice, and mental health (but not general practice nursing), the number of clinically important articles was correlated withScience Citation Index (SCI) Impact Factors.

**Conclusions:**

Although many clinical journals publish high-quality, clinically relevant and important original studies and systematic reviews, the articles for each discipline studied were concentrated in a small subset of journals. This subset varied according to healthcare discipline; however, many of the important articles for all disciplines in this study were published in broad-based healthcare journals rather than subspecialty or discipline-specific journals.

## Background

Evidence on the journal-reading habits of clinicians comes from three separate groups of publications. First, several surveys have been used to ascertain the reading habits of physicians. Fafard and Snell [1] assessed house staff who reported reading an average of 8.7 hours per week, with about half of their time spent reading for specific patient situations. Reading time for family practice residents was more than three hours per week [2,3] and ranged from 1–12 hours. Dermatology residents averaged 4.2 hours reading per week and read an average of seven journals, four of which were peer reviewed [4]. Internists read an average of 4.4 hours per week [3], while surgeons reported an average reading time of 3.5 hours across 3–16 journals [5]. This average of three-four hours of reading time per week is quite consistent across disciplines, level of education, time, and nationality.

A second set of surveys and studies center on general information-seeking behaviors of clinicians. These studies show how journal reading fits in with the other types of information that clinicians use. Two systematic reviews have been done recently.

Researchers at the Australian National Institute of Clinical Studies [6] reviewed preferred information sources in many clinician groups, including physicians (primary care/general practice/family practice, hospitalists, rural physicians, diabetologists); nurses (hospital and occupational health nurses); physical therapists; dental hygienists; and policy-makers. They reviewed 34 studies and concluded that all groups used multiple information resources, with information needs answered most often by other people, followed by books and journals. Dawes and Sampson [7] evaluated 19 studies of physician information-seeking behavior. They placed books and journals in one category (print resources) and found this to be the most used information source, with colleagues being the second.

The third source of information on clinicians' use of information resources comes from marketing studies. The Association of Medical Publications [8] monitors physician use of printed journals and other information resources. Despite the rapid expansion of the Internet and all the information it contains, physicians continue to read and value journal articles, and their reliance on journals may be increasing. Data collected in 1983 and 1998 shows that physician reliance on journal literature as their main source of medical information increased from 61.8% to 76.3%, an absolute increase of 14.5% in 15 years.

The importance of reading journal articles for clinical care is evident. The increasing number of journals from which important and relevant articles are found, combined with the decreasing number of personal subscriptions [9], makes it more important than ever for physicians to choose carefully which journals to subscribe to and read. This decision should not be based on intuition alone, as shown in an important study by obstetricians and gynecologists. Weiner *et al. *[10] sought to determine which journals had published numerical data on the relation between oral contraceptive use and cancer–information they judged to be clinically important and readily available in their subspecialty journals. Assessing 3735 articles identified by MEDLINE searches, only 27 studies reported numerical data, of which 23 were published in mainstream general medical journals. Only four were published in obstetrics and gynecology journals.

Since the publication of the study by Weiner *et al. *[10], several groups have tried to determine targeted journal subsets that could provide the most important clinical information to physicians in different specialties. Birken and Parkin [11] assessed journals with pediatric content. Using data from pediatric-related systematic reviews in the *Cochrane Database for Systematic Reviews *for 1997, as well as policy statements from the American Academy of Pediatricians and the Canadian Paediatric Society, they determined that four general medical journals and three pediatric specialty journals provided access to most of the important advances: *Archives of Diseases in Childhood, BMJ, JAMA, Journal of Pediatrics, Lancet, New England Journal of Medicine, *and *Pediatrics*. Their results validate the findings of Weiner *et al.*–important studies in a discipline or specialty are often not published in specialty journals.

Gehanno and Thirion [12] used MEDLINE searches and the Science Citation Index (SCI) Impact Factors to identify journal subsets in occupational health. Eight journals provided coverage of 27% their discipline content; 38 journals increased this to 52%. Coverage needed to be expanded beyond their specialty journals for them to remain current in occupational health.

Lee *et al. *[13] sampled research articles from 30 randomly selected journals from a list of 107 general internal medicine journals defined by SCI. They found that journals with high citation rates, SCI Impact Factors, and circulation rates; low manuscript acceptance rates; and listing on the Brandon/Hill Library List [14] were predictive of higher article methodologic scores.

Ebell *et al. *[15] as well as our research group [16] present an alternative approach for clinicians to keep up to date with current literature. Both groups produce summaries of important advances in areas of clinical care so that individuals do not have to read primary journals and evaluate reports. Ebell *et al. *provided results of a hand search of 85 core journals of interest to family/general practice. Physicians read these journals for six months and identified articles that were considered to be POEMs (patient-oriented evidence that matters). A POEM addresses a clinical question encountered by a family physician at least once every two weeks, measures patient-oriented outcomes, and presents results that will likely affect practice. The report provides summaries of which journals publish important clinical advances for general/family practice.

In this article we report on a survey of the contents of 170 core clinical journals for the publishing year 2000 to assess which journals publish the highest number of methodologically sound and clinically relevant studies in the disciplines of internal medicine, general/family practice, general practice nursing, and mental health. In the "Methods" section we describe our two-step article selection process for clinical importance and methodologic rigor, which is very similar to that used by Ebell *et al. *[15]. The data we provide reflects the merit of individual journal titles from a clinical perspective; it may help clinicians to choose which journals to read, and health sciences libraries to include them in their collections.

## Methods

The Health Information Research Unit of the Department of Clinical Epidemiology and Biostatistics at McMaster University, Ontario, Canada, publishes several secondary "evidence-based" journals, systematically selecting, summarizing and appraising articles in a broad range of primary clinical journals. In 2000 we prepared *ACP Journal Club (ACP J Club) *to support internal medicine , *Evidence-Based Medicine (EBM) *to support general/family practice, *Evidence-Based Nursing (EBN) *to support general care nursing, and *Evidence-Based Mental Health (EBMH) *to support mental health. To identify potential candidate articles for inclusion in these journals, six Masters-level trained staff read each article in the major general healthcare journals and those in the disciplines and subdisciplines related to the content of each abstract journal. The list of these journals (see Additional File 1) is comprised of titles suggested by librarians, clinicians, editors, and editorial staff; SCI Impact Factors; and systematic examination of the contents of each title for at least six months. More than 400 journal titles have been assessed since the abstract journals were started in 1991.

We consider the *Cochrane Database of Systematic Reviews *to be a separate journal that publishes systematic reviews of the literature on a quarterly basis. This is consistent with the U.S. National Library of Medicine's decision to index the *Cochrane Database of Systematic Reviews *as a separate journal. We evaluate only the new reviews and those that are substantially updated each quarter. We do not consider the rest of the database or protocols that describe reviews that are in progress or being planned.

Original and review articles are placed in one or more of seven categories of study type–therapy and prevention, screening and diagnosis, prognosis, etiology and harm, economics and cost, clinical prediction guides, and qualitative studies [16]. All categories have a set of pass/fail rules for selection (see: ), except for qualitative and cost studies. Basic inclusion criteria are that the articles i) are about the healthcare of humans; ii) have at least one clinically important outcome; and iii) use appropriate statistical analyses. As an example of category-specific criteria, an article on screening or diagnosis must meet these additional criteria:

• a spectrum of participants were included, some with the disease or condition of interest and some without

• objective diagnoses were made using the "gold" standard or current clinical standard for diagnosis of the disease or condition

• participants received both the new test and some form of the diagnostic standard

• the diagnostic standard was interpreted without knowledge of the test result and vice versa.

The basic inclusion criteria are based on study design and methodology principles for evidence-based healthcare. Their use identifies studies that have data related to patients or those at risk of disease, diseases and conditions, and real-life clinical settings. Therefore, a study or review article that meets the criteria can be considered to be appropriate for possible use in patient care decision-making. The article readers are trained and retested annually so that they can reliably apply these selection rules for inclusion in our evidence-based journals (kappa measuring chance-adjusted agreement > 80% for all categories) [16].

With a research grant from the U.S. National Library of Medicine we intensified our data collection from the reading process related to the evidence-based journals for the publishing year 2000. All articles in 170 journals were classified as to whether they were "of interest" to the healthcare of humans and, if so, whether they reported original data or were systematic review articles. These original studies and reviews were classified into all possible categories (where more than one category could apply, for example, a therapy article that included economic data), and were then given a pass or fail methodologic designation for each category.

Articles passing methodologic criteria were assessed further for clinical interest by an editorial group of practicing clinicians for each abstract journal. These clinicians have expertise in methodology and specific areas of healthcare such as gastroenterology or neonatology nursing. At this point the clinician raters excluded all studies with preliminary results, interventions that were not readily available or proven useful, already known and applied findings, and topics addressing rare conditions or diseases. After review, often by a team of three-five clinicians (see  for a copy of the rating system that was used in paper format for this study) some articles were further processed. The editors chose articles to be abstracted that they considered to have the most important message for clinicians. The remaining pass articles were listed as "Other Articles Noted" if their content was of relevance to the disciplines covered by the abstract journals.

This dual selection process (methodologic rigor and clinical importance) provided insight into which journals yielded the highest numbers of pass articles. This had major implications for clinical practice at two levels of clinical relevance. The more stringent level includes articles that were summarized in each abstract journal. The second, less stringent level includes articles that are abstracted as well as those articles that are listed in the Other Articles Noted sections. Analysis was done by abstract journal title (*ACP J Club, EBM, EBN*, and *EBMH*) to ascertain which journal titles were most important to their target clinical audience (internal medicine, general/family practice, general practice nursing, and mental health, respectively). SCI Impact Factors were collected for each journal title for each discipline. If an SCI Impact Factor was not available we sought Social Science Index Impact Factors. These data were analyzed to determine if Impact Factors were related to yield of clinically important advances, as found by Lee *et al. *[13] and Gehanno and Thiron [12].

## Results

For 2000, the 170 core journals we selected published 60,352 articles. The total number of pass articles was 3059 for original studies and 1073 for reviews. An article could be counted more than once if it passed for multiple categories. Six journals did not publish any pass articles. The complete list of journals and their yield appears in the Additional File 1.

The category breakdown of pass articles for original studies and review articles, respectively, was 1639 and 662 for therapy and prevention, 152 and 47 for screening and diagnosis, 195 and 22 for prognosis, 290 and 308 for etiology and harm, 35 and 10 for economics, 358 and 8 for qualitative studies, and 93 and 4 for clinical prediction guides.

The top 20 journals for yield of pass articles are included in Table 1. The titles varied considerably in both the total number and proportion of clinically relevant articles that they published. For example, 95.0% of the articles (all reviews) in the *Cochrane Database of Systematic Reviews *passed our criteria, while only 2.8% of the articles in the *American Journal of Gastroenterology *met standards for clinically applicable studies. (The *American Journal of Gastroenterology *is a specialty journal and a substantial proportion of its content is preclinical. These preclinical articles, by definition, did not meet the clinical criteria in this study.) Generally, a clinical reader would need to read in the range of 13–14 articles from these top 20 journals to obtain one that is directly clinically important in any healthcare area, although the range is substantial (1.1 to 36.9). We call this number the "number of articles needed to be read" or NNR.

The number of pass articles did not correlate with SCI Impact Factors for the top 20 journals (correlation coefficient 0.29, *P *= 0.24). Analysis of the top 50 titles showed a weak correlation (correlation coefficient 0.41, *P *= 0.004) for the same analysis.

The breakdown by discipline was done using the total number of articles that were selected for inclusion in each of the four abstract journals–internal medicine, general/family practice, general care nursing, and mental health (Tables 2,3,4,5). Both the total number of articles abstracted and the total number of articles in each journal (abstracted and "Other Articles Noted") are included in Tables 2, 4, and 5 giving a two-level assessment of "clinical worth". *EBM *does not publish an "Other Articles Noted" Section.

### Internal medicine content (ACP J Club)

The journals contributing articles important to the practice of internal medicine (*ACP J Club) *are shown in Table 2. Substantial drop-off is seen after the top three titles (*New England Journal of Medicine, JAMA, *and *Lancet)*. These three journals and the *Cochrane Database of Systematic Reviews *provided 56.5% of the articles abstracted, with 28 additional journals providing the other 43.5%. Fifteen titles provided only one article each and, overall, 32 journals provided at least one article for abstraction. Another 51 journals provided at least one article in the "Other Articles Noted" section. Thus, 83 journals from our list of 170 published studies important to internal medicine.

The NNR to obtain one high-quality and clinically relevant study or review varied considerably across the titles. For the more stringent definition of clinical relevance (article abstracted in *ACP J Club)*, the range of NNR for internal medicine was from 40.4 for the *Cochrane Database of Systematic Reviews *to 1334 for *Neurology*. For the less stringent definition (article abstracted or noted in *ACP J Club), *the NNR range for internal medicine was from 3.4 for the *Cochrane Database of Systematic Reviews *to 242 for *Acta Obstetrica et Gynaecologica Scandinavica*.

Correlating the number of articles published in *ACP J Club *with their SCI Impact Factor showed a large and positive correlation for both levels of clinical importance (correlation coefficient 0.786, *P *< 0.001 for the more stringent definition; correlation coefficient 0.688, *P *< 0.001 for the less stringent definition of clinical importance). These findings support the findings by Lee *et al. *[13] that SCI Impact Factors were correlated with quality articles for general internal medicine.

### General/family practice content (EBM)

The most important articles for general/family practice (publication in *EBM*) were published in *BMJ, Lancet, Cochrane Database of Systematic Reviews, Archives of Disease in Childhood*, and *Annals of Internal Medicine*–these journals provided 55.6% of *EBM *content (Table 3). Overall, 45 titles provided abstracts for general/family practice coverage. The "shape" of the data is different for general/family practice than for general internal medicine, with more journals providing articles for abstraction. This is consistent with the discipline because general/family practitioners must use knowledge from a broader range of health conditions (including pediatrics and obstetrics, for example) than general internists and other specialists. Only the most stringent definition for clinical worth could be evaluated for *EBM *content because the "Other Articles Noted" section of the journal did not exist in the year 2000. The NNR for general/family practice ranged from 55 for the *Cochrane Database of Systematic Reviews *to 1351 for *Circulation*.

Correlation analysis showed that the number of qualified articles in each journal title was associated with the journal's SCI Impact Factor (correlation coefficient 0.546, *P *= < 0.001). This shows substantial agreement between SCI Impact Factors and number of articles but slightly less agreement than that found using the general internal medicine data (correlation coefficients > 0.688).

### General care nursing content (EBN)

Nursing content came from many journals, including journals that are considered to be primarily targeted at physicians, and was not concentrated in a small set of journal titles (Table 4). To reach 51.0% of the abstracted articles, seven titles were needed (*Qualitative Health Research, Cochrane Database of Systematic Reviews, Pediatrics, JAMA, Lancet, BMJ*, and *Journal of Advanced Nursing*). Thirty-two other journals provided articles for abstraction and 33 journals provided studies that were listed only in the "Other Articles Noted" section; 72 journals in total provided content for general care nursing. The NNR for general practice nursing was variable, ranging from 6.0 for *Qualitative Health Research *to 1530 for *New England Journal of Medicine *for the more stringent definition of clinical relevance. For the less stringent definition of clinical relevance the NNRs ranged from 4.7 for *Qualitative Health Research *to 923 for *American Journal of Gastroenterology*. The low NNR for *Qualitative Health Research *undoubtedly reflects the fact that only clinical criteria for relevance were applied in the selection of qualitative studies, not explicit methodologic criteria. The reason for lack of methodologic criteria for qualitative studies was that we have been unable to obtain agreement from qualitative researchers of what the quality criteria should be.

No correlation was seen between number of articles published per journal title and SCI Impact Factors for either the stringent definition of clinical relevance (correlation coefficient 0.096, *P *= 0.57) or the less strict definition (correlation coefficient 0.256, *P *= 0.038).

### Mental health content (EBMH)

Mental health content was also spread over a broader range of journals than was internal medicine (Table 5). To reach 53.2% of the articles abstracted, nine titles needed to be read: *Archives of General Psychiatry, Cochrane Database of Systematic Reviews, American Journal of Psychiatry, British Journal of Psychiatry, JAMA, Lancet, International Journal of General Psychiatry, Journal of the American Academy of Child and Adolescent Psychiatry, *and *Journal of Consulting and Clinical Psychology. *Forty-one titles provided at least one article for abstraction. The titles in Table 5 show that studies related to mental health are published in many journals and specialties–a reflection of the broad nature of the discipline. The NNR for mental health for the most stringent definition of clinical relevance ranged from 20.1 for *Archives of General Psychiatry *to 1142.7 for *BMJ*. *Archives of General Psychiatry *also has the lowest NNR for the less stringent definition (11.5), with *CMAJ *having the highest NNR (1007) of those journals with at least one article on mental health. *EBMH *has a smaller "Other Articles Noted" section. Only eight additional journals provide articles for this section beyond the 61 that provide articles for abstraction.

A weak association was shown between the number of published mental health articles and SCI Impact Factors (correlation coefficient 0.386, *P *= 0.02 for the more stringent definition; and correlation coefficient 0.381, *P *= 0.01 for the less stringent definition).

### All disciplines

Combining the content across the four discipline areas, we again see the concentration of important clinically relevant articles in a small subset of journals. Eight journals provided at least one article for abstraction to all four abstract journals: *Annals of Internal Medicine, Archives of Internal Medicine, BMJ, CMAJ, Cochrane Database of Systematic Reviews, JAMA, Lancet, *and *New England Journal of Medicine. *Another 10 journals provided at least one article to three of the four abstract journals: *American Journal of Medicine, Archives of General Psychiatry, British Journal of General Practice, British Journal of Surgery, Health Psychology, Journal of Clinical Epidemiology, Journal of Clinical Psychopharmacology, Journal of Family Practice, Journal of the American Geriatrics Society, *and *Pediatrics. *Twenty-eight journals provided studies to two of the abstract journals, 36 provided articles to at least one abstract journal, and 82 titles provided no articles for abstraction (excluding the six titles that did not publish any pass articles).

## Conclusions

We found that the majority of articles for each discipline were sequestered in a small subset of journals. This is consistent with Bradford's Law of Scattering for journal subsets, which states that the important articles on any topic will be concentrated in a small subset of journals with exponential drop-off in numbers of relevant articles across journal titles [17]. Across disciplines and study areas, approximately 70% of articles are often found in 30% of journals in any given area of study.

Not surprisingly, for broad-based disciplines such as mental health and nursing, the number of titles was greater than for more focused disciplines such as internal medicine. SCI Impact Factors were highly correlated with the number of important clinical articles in separate titles for internal medicine and, to a lesser extent, for general/family practice, and mental health but not for general practice nursing. This likely reflects the volume of clinically important research activity in these fields–with especially high volumes in the disorders managed by internal medicine and its subspecialties–coupled with the avidity of authors from all disciplines to submit their best studies to the high-circulation general journals.

As found by Weiner *et al. *[10] and others, most of the important advances in any discipline are not published in specialty journals but in the more general healthcare journals such as *JAMA, Lancet, BMJ, New England Journal of Medicine, *and *Cochrane Database of Systematic Reviews*. Health professionals in all disciplines should be aware that major advances in any field will most likely be published in the main general medicine journals, while at the same time recognizing that specialty journals also publish important information. Much variation exists across journal titles in both the number and proportion of articles that are high quality, clinically important, and newsworthy. Variation also exists across disciplines. It is also interesting to note that all lists of important journals discussed in this report and also the one by Ebell *et al. *[15] include both North American and European titles. Reading choices for clinicians cannot be based on national or discipline boundaries alone.

Of the 45 titles that provided articles to *EBM*, 23 were on the list provided by Ebell *et al. *(POEM articles) [15]. Ebell *et al. *found common POEMs in 49 journals and any POEMs in 64 journals. POEMs and *EBM *cover the content of general/family practice by considering a similar number of journals, although both groups read approximately 50% unique titles. Ebell *et al. *read 85 titles for POEMs articles and we read 170 titles for this study. Our coverage of clinical content was broader and included internal medicine, general practice nursing, and mental health, but 53 titles were read by both groups. Correlational analysis for the ranking of each journal title according to the number of articles identified as clinically important showed a small but significant agreement (0.4397, *P *= 0.005) when comparing our list with the list by Ebell *et al.*

Consistent with the data from Weiner *et al. *[10], many advances important to general practice nursing are not published in nursing specialty or discipline-specific journals. Only four of the top 17 and eight of the top 41 journals in Table 4 are considered nursing specialty titles. Overall 39 titles provided at least one article for abstraction and an additional 33 titles provided at least one article to the "Other Articles Noted" section, again showing the broader spectrum of journals that publish articles important to general care nursing.

Clinicians in the target disciplines described here could use our findings to focus their fulltext readings. For other disciplines, a similar audit of clinical yield would be needed, either from an appropriate secondary journal that systematically reviews specified journals, or an independent audit. Another approach to staying current may be to subscribe to one or more secondary journals that highlight important clinical advances. These secondary publications have not only selected the most appropriate studies for clinical consideration, they highlight important aspects of methodology and implementation. This assessment of studies before application can be time-consuming and difficult for many clinicians, and involves a certain amount of training and practice to become proficient. Many examples of secondary publications exist in various disciplines and include the four studied in this report, POEMs [15], and *Journal Watch*. Use of these summaries of studies and reviews can be supplemented by access to fulltext articles.

Many academic medical centers and hospitals provide good online access to major healthcare journals. For example, the Health Sciences Library of the University of Pittsburgh, PA, USA, provides online access to 24 of the top 25 journals in this study and all 25 of the journals identified as high yielders by Ebell *et al. *[15]. Specialized health libraries with limited budgets may wish to focus on the journals, either in paper or electronic format, with the highest yield for the disciplines they serve.

## List of abbreviations

ACP J Club *ACP Journal Club *(journal)

AHCPR Agency for Health Care Policy and Research (now AHRQ)

AHRQ Agency for Healthcare Research and Quality (formerly AHCPR)

CCOHTA Canada Coordinating Office for Health Technology Assessment

EBM *Evidence-Based Medicine *(journal)

EBMH *Evidence-Based Mental Health *(journal)

EBN *Evidence-Based Nursing *(journal)

NNR Number of articles needed to read to obtain one high-quality and clinically relevant study or review

POEM Patient-oriented evidence that matters

SCI Science Citation Index

## Competing interests

The authors all worked with *ACP Journal Club, Evidence-Based Medicine, Evidence-Based Nursing, *and *Evidence-Based Mental Health *at the time of this study, and were paid for this work, but the publishers of these journals were not involved in the study, which was funded externally. The authors do not hold stocks or shares in any company that may benefit from the publication of this paper.

## Author contributions

NLW and RBH prepared grant submissions in relation to this project. All authors drafted and commented on the manuscript and approved the final manuscript, as well as supplied intellectual content to the collection and analysis of the data. NLW and KAM did data collection and analysis, and supervised research staff.

## Pre-publication history

The pre-publication history for this paper can be accessed here:



## Supplementary Material

Additional File 1 Additional File 1 includes a list of the 170 journals read for 2000 along with the number of articles reviewed, the number and percentage that passed criteria, and the NNR (number of articles that are needed to be read to obtain one that is clinically relevant and has high-quality methods). The file name is "Publishing Important Articles Appendix.doc" and it is in Word 2000 format. Click here for file

## Figures and Tables

**Table 1:** Number of high-quality, clinically relevant articles in the top 20 clinical journals for 2000

**Journal title**	**Number of articles with abstracts (from MEDLINE)**	**Number of articles evaluated**	**Number of pass articles / number evaluated (% pass)**	NNR* for number evaluated	SCI Impact Factor** for 2000
*Cochrane Database of Systematic Reviews*	1004	444	422 (95.0)	1.1	Not available
***Lancet***	**669**	**3858**	**134 (3.5)**	**28.8**	**10.2**
*Journal of Clinical Oncology*	445	650	100 (15.4)	6.6	8.8
***BMJ***	**209**	**3428**	**93 (2.7)**	**36.9**	**5.3**
*Circulation*	925	1351	92 (6.8)	14.7	10.9
***Journal of Advanced Nursing***	**341**	**611**	**92 (15.1)**	**6.6**	**0.77**
*Obstetrics and Gynecology*	389	478	88 (18.4)	5.4	2.0
***JAMA***	**329**	**1930**	**87 (4.5)**	**22.2**	**16.4**
*New England Journal of Medicine*	228	1530	83 (5.4)	18.4	29.5
***Archives of Internal Medicine***	**340**	**620**	**81 (13.1)**	**7.7**	**6.1**
*Journal of the American College of Cardiology*	514	707	76 (10.7)	9.3	7.1
***Pediatrics***	**548**	**811**	**76 (9.4)**	**10.7**	**4.8**
*American Journal of Cardiology*	631	850	72 (8.5)	11.6	2.7
***American Journal of Obstetrics and Gynecology***	**539**	**704**	**72 (10.2)**	**9.8**	**2.5**
*Critical Care Medicine*	340	977	70 (7.2)	14.0	3.8
***Chest***	**589**	**882**	**66 (7.5)**	**13.4**	**2.5**
*Stroke*	402	609	59 (9.7)	10.3	6.0
***Neurology***	**814**	**1334**	**58 (4.3)**	**23.0**	**4.8**
*American Journal of Gastroenterology*	474	923	56 (2.8)	16.6	
***Diabetes Care***	**263**	**529**	**55 (10.4)**	**9.6**	**5.0**
Average			7.3%	13.8	

*The NNR is the number of articles that would have to be read in each journal to identify one with high quality methods that is clinically relevant; **The SCI Impact Factor is the Science Citation Index Impact Factor (rating of how important each journal is in relation to citations). Data are for 2000. Articles have not been screened for direct clinical relevance beyond basic criteria of having at least one clinically important outcome.

**Table 2:** *ACP J Club *(internal medicine) journal-specific content of high-quality, clinically relevant articles

**Journal title**	**Number articles reviewed in 2000**	**Number abstracted (% included in *ACP J Club*)**	**NNR for abstracted article**	Number abstracted or listed (% included in *ACP J Club*)*	NNR for abstracted or listed**
*New England Journal of Medicine*	1530	25 (16.9)	61.2	67 (6.7)	22.8
***JAMA***	**1930**	**25 (16.9)**	**77.2**	**53 (5.4)**	**36.4**
*Lancet*	3858	22 (14.9)	175.4	62 (6.6)	62.2
Cochrane Database Systematic Reviews***	**444**	**11 (7.4)**	**40.4**	**130 (13.1)**	**3.4**
*Annals of Internal Medicine*	602	8 (5.4)	75.3	33 (3.3)	18.2
***Archives of Internal Medicine***	**620**	**6 (4.1)**	**103.3**	**57 (5.8)**	**24.8**
*BMJ*	3428	5 (3.4)	685.6	50 (5.1)	68.6
***Circulation***	**1351**	**5 (3.4)**	**270.2**	**33 (3.3)**	**40.9**
AHRQ/AHCPR Reports***	N.A.	4 (2.7)	N.A.	9 (1.0)	N.A.
***American Journal of Gastroenterology***	**923**	**4 (2.7)**	**225.6**	**21 (2.1)**	**44.0**
*American Journal of Medicine*	435	3 (2.0)	130.5	21 (2.1)	20.7
***CMAJ (formerly Canadian Medical Association Journal)***	**1007**	**3 (2.0)**	**335.7**	**12 (1.2)**	**83.9**
*Diabetic Medicine*	188	3 (2.0)	62.6	13 (1.3)	14.5
***Thorax***	**336**	**3 (2.0)**	**112.0**	**8 (0.8)**	**42.0**
*Annals of Emergency Medicine*	294	2 (1.4)	147.0	11 (1.1)	26.7
***Journal of the American Geriatrics Society***	**384**	**2 (1.4)**	**192.0**	**18 (1.8)**	**21.3**
*Journal of Vascular Surgery*	544	2 (1.4)	272.0	6 (0.6)	9.0
***American Journal of Cardiology ***	**850**	**1 (0.7)**	**850.0**	**14 (1.4)**	**60.7**
*Archives of Neurology*	313	1 (0.7)	313	2 (0.2)	156.5
***British Journal of Surgery***	**402**	**1 (0.7)**	**402**	**6 (0.6)**	**67.0**
CCOHTA Reports****	N.A.	1 (0.7)	N.A.	1 (0.1)	N.A.
***Critical Care Medicine***	**977**	**1 (0.7)**	**977**	**24 (2.4)**	**40.7**
*Diabetes Care*	529	1 (0.7)	529	14 (1.4)	37.8
***Gastroenterology***	**543**	**1 (0.7)**	**543**	**5 (0.5)**	**108.5**
*Gut*	446	1 (0.7)	446	6 (0.6)	74.3
***Health Psychology***	**79**	**1 (0.7)**	**79**	**3 (0.3)**	**26.6**
*Journal of Clinical Psychopharmacology*	162	1 (0.7)	162	1 (0.1)	81.0
***Journal of Family Practice***	**263**	**1 (0.7)**	**263**	**10 (1.0)**	**26.3**
*Journal of Infectious Diseases*	760	1 (0.7)	760	6 (0.6)	126.7
***Neurology***	**1334**	**1 (0.7)**	**1334**	**12 (1.2)**	**111.2**
*Spine*	604	1 (0.7)	604	14 (1.4)	43.1
***Stroke***	**609**	**1 (0.7)**	**609**	**26 (2.6)**	**101.5**
*Acta Obstetrica et Gynecologica Scandinavica*	242	0 (0.0)		1 (0.1)	242.0
***Addiction***	**295**	**0 (0.0)**	**Infinity**	**4 (0.4)**	**63.8**
*Age and Ageing*	767	0 (0.0)	Infinity	6 (0.6)	127.8
***Alimentary Pharmacology and Therapeutics***	**N.A.**	**0 (0.0)**	**N.A.**	**4 (0.4)**	**N.A.**
*American Journal of Epidemiology*	362	0 (0.0)	Infinity	18 (1.8)	20.1
***American Journal of Obstetrics and Gynecology***	**704**	**0 (0.0)**	**Infinity**	**2 (0.2)**	**352.20**
*American Journal of Psychiatry*	508	0 (0.0)	Infinity	1 (0.1)	508
***American Journal of Public Health***	**363**	**0 (0.0)**	**Infinity**	**5 (0.5)**	**72.6**
*American Journal of Respiratory and Critical Care Medicine*	783	0 (0.0)	Infinity	9 (0.9)	87.0
***Annals of Rheumatic Diseases***	**266**	**0 (0.0)**	**Infinity**	**1 (0.1)**	**266.0**
*Annals of Surgery*	301	0 (0.0)	Infinity	3 (0.3)	100.3
***Archives of Family Medicine (no longer published)***	**230**	**0 (0.0)**	**Infinity**	**6 (0.6)**	**38.3**
*Archives of General Psychiatry*	161	0 (0.0)	Infinity	2 (0.2)	80.5
***Archives of Physical Medicine and Rehabilitation***	**337**	**0 (0.0)**	**Infinity**	**4 (0.4)**	**84.3**
*Archives of Surgery*	330	0 (0.0)	Infinity	3 (0.3)	103.3
***Australian and New Zealand Journal of Psychiatry***	**214**	**0 (0.0)**	**Infinity**	**1 (0.1)**	**214.0**
*British Journal of General Practice*	453	0 (0.0)	Infinity	3 (0.3)	151
***British Journal of Psychiatry***	**402**	**0 (0.0)**	**Infinity**	**2 (0.2)**	**201**
*Canadian Journal of Gastroenterology*	145	0 (0.0)	Infinity	3 (0.3)	48.3
***Canadian Journal of Infection Control***	**31**	**0 (0.0)**	**Infinity**	**1 (0.1)**	**31.0**
*Canadian Respiratory Journal*	68	0 (0.0)	Infinity	2 (0.2)	34.0
***Cancer***	**786**	**0 (0.0)**	**Infinity**	**3 (0.3)**	**262.0**
*Chest*	882	0 (0.0)	Infinity	21 (2.1)	42.0
***Heart***	**450**	**0 (0.0)**	**Infinity**	**6 (0.6)**	**75.0**
*Heart and Lung*	59	0 (0.0)	Infinity	1 (0.1)	59.0
***Hypertension***	**419**	**0 (0.0)**	**Infinity**	**6 (0.6)**	**69.8**
*International Journal of Geriatric Psychiatry*	169	0 (0.0)	Infinity	6 (0.6)	28.2
***Journal of Affective Disorders***	**154**	**0 (0.0)**	**Infinity**	**3 (0.3)**	**51.3**
*Journal of the American Board of Family Practice*	121	0 (0.0)	Infinity	1 (0.1)	121.0
***Journal of the American College of Cardiology***	**707**	**0 (0.0)**	**Infinity**	**29 (2.9)**	**24.4**
*Journal of Bone and Joint Surgery (US)*	360	0 (0.0)	Infinity	1 (0.1)	360.0
***Journal of Clinical Epidemiology***	**173**	**0 (0.0)**	**Infinity**	**8 (0.8)**	**21.6**
*Journal of Epidemiology and Community Health*	205	0 (0.0)	Infinity	6 (0.6)	34.2
***Journal of General Internal Medicine***	**155**	**0 (0.0)**	**Infinity**	**9 (0.9)**	**17.2**
*Journal of Internal Medicine*	177	0 (0.0)	Infinity	4 (0.4)	44.3
***Journal of Neurology and Neurosurgery and Psychiatry***	**478**	**0 (0.0)**	**Infinity**	**7 (0.7)**	**68.3**
*Journal of Psychosomatic Research*	118	0 (0.0)	Infinity	6 (0.6)	19.7
***Journal of Rheumatology***	**657**	**0 (0.0)**	**Infinity**	**4 (0.4)**	**164.3**
*Journal of Trauma Injury Infection and Critical Care*	562	0 (0.0)	Infinity	2 (0.2)	281.0
***Medical Care***	**162**	**0 (0.0)**	**Infinity**	**7 (0.7)**	**32.1**
*Medical Journal of Australia*	598	0 (0.0)	Infinity	1 (0.1)	598.0
***Pain***	**269**	**0 (0.0)**	**Infinity**	**7 (0.7)**	**38.4**
*Patient Education and Counseling*	94	0 (0.0)	Infinity	1 (0.1)	13.3
***Pediatrics***	**811**	**0 (0.0)**	**Infinity**	**2 (0.2)**	**405.5**
*Psychology and Aging*	55	0 (0.0)	Infinity	1 (0.1)	55.0
***Psychological Medicine***	**142**	**0 (0.0)**	**Infinity**	**4 (0.4)**	**35.3**
*Psychosomatic Medicine*	106	0 (0.0)	Infinity	1 (0.1)	106.0
***Qualitative Health Research***	**60**	**0 (0.0)**	**Infinity**	**1 (0.1)**	**60.0**
*Radiology*	654	0 (0.0)	Infinity	1 (0.1)	654.0
***Rheumatology***	**339**	**0 (0.0)**	**Infinity**	**9 (0.9)**	**37.7**
*Social Science and Medicine*	302	0 (0.0)	Infinity	2 (0.2)	151.0
***Western Journal of Nursing Research***	**99**	**0 (0.0)**	**Infinity**	**1 (0.1)**	**99.0**
*TOTAL*		148 (100)		990 (100)	
*Correlation with SCI Impact Factors-correlation coefficient (P-value)*	N.A.	0.788 (< 0. 001)	N.A.	0.688 (< 0.001)	N.A.

*Articles abstracted are those that pass methodological criteria and are deemed to be the most important by practicing internists. The other articles listed are articles with the same high-quality methods but are considered to be slightly less important clinically by practicing clinicians; **The number of articles needed to read (NNR) is a measure of the ratio of number of relevant articles (abstracted or combined abstracted or listed) divided into the total number of articles for each journal title; ***SCI Impact Factors not available for analysis; ****Canada Coordinating Office for Health Technology Assessment Reports; N.A. Not applicable. Data are for 2000. Note that the AHRQ/AHCPR and CCOHTA reports were not considered journal titles and read as such for this report.

**Table 3:** *EBM *(general/family practice) journal-specific content of high-quality, clinically relevant articles^†^

**Journal title**	**Number articles in 2000 in journal**	**Number abstracted (% included in *EBM*)**	**NNR for abstracted article**	Number abstracted or listed (% included in *EBM*)*	NNR for abstracted or listed**
*JAMA*	1930	18 (12.5)	107.2	N.A.	N.A.
***BMJ***	**3428**	**17 (11.8)**	**201.6**	**N.A.**	**N.A.**
*Lancet*	3858	17 (11.8)	226.9	N.A.	N.A.
***New England Journal of Medicine***	**1530**	**13 (9.0)**	**117.6**	**N.A.**	**N.A.**
Cochrane Database of Systematic Reviews***	444	8 (5.6)	55.0	N.A.	N.A.
***Annals of Internal Medicine***	**602**	**7 (4.9)**	**86.1**	**N.A.**	**N.A.**
AHRQ/AHCPR Reports***	--	6 (4.2)	N.A.	N.A.	N.A.
***Archives of Disease in Childhood***	**392**	**4 (2.8)**	**98.0**	**N.A.**	**N.A.**
*American Journal of Medicine*	435	4 (2.8)	108.8	N.A.	N.A.
***Archives of Family Medicine (no longer published)***	**230**	**3 (2.1)**	**76.7**	**N.A.**	**N.A.**
*Journal of Family Practice*	263	3 (2.1)	87.7	N.A.	N.A.
***Annals of Emergency Medicine***	**294**	**2 (1.4)**	**147.0**	**N.A.**	**N.A.**
*BJOG (formerly British Journal of Obstetrics and Gynaecology)*	334	2 (1.4)	157.0	N.A.	N.A.
***British Journal of Psychiatry***	**335**	**2 (1.4)**	**167.5**	**N.A.**	**N.A.**
*Journal of Pediatrics*	444	2 (1.4)	222.0	N.A.	N.A.
***American Journal of Gastroenterology***	**923**	**2 (1.4)**	**461.5**	**N.A.**	**N.A.**
*British Journal of Surgery*	402	2 (1.4)	201	N.A.	N.A.
***Diabetic Medicine***	**188**	**2 (1.4)**	**144**	**N.A.**	**N.A.**
*Journal of Vascular Surgery*	544	2 (1.4)	272	N.A.	N.A.
***Neurology***	**1334**	**2 (1.4)**	**667.0**	**N.A.**	**N.A.**
*Pediatrics*	811	2 (1.4)	405.5	N.A.	N.A.
***Annals of Surgery***	**301**	**1 (0.7)**	**301**	**N.A.**	**N.A.**
*Archives of General Psychiatry*	161	1 (0.7)		N.A.	N.A.
***Archives of Internal Medicine***	**620**	**1 (0.7)**	**620**	**N.A.**	**N.A.**
*Archives of Neurology*	313	1 (0.7)	313	N.A.	N.A.
***Archives of Pediatric and Adolescent Medicine***	**273**	**1 (0.7)**	**273**	**N.A.**	**N.A.**
*Arthritis and Rheumatology*	440	1 (0.7)	440	N.A.	N.A.
***British Journal of General Practice***	**453**	**1 (0.7)**	**453**	**N.A.**	**N.A.**
*Canadian Respiratory Journal*	68	1 (0.7)	68	N.A.	N.A.
***Circulation***	**1351**	**1 (0.7)**	**1351**	**N.A.**	**N.A.**
*CMAJ (formerly Canadian Medical Association Journal)*	1007	1 (0.7)	1007	N.A.	N.A.
***Diabetes Care***	**529**	**1 (0.7)**	**529**	**N.A.**	**N.A.**
*Gastroenterology*	543	1 (0.7)	543	N.A.	N.A.
***Gut***	**446**	**1 (0.7)**	**446**	**N.A.**	**N.A.**
*Health Psychology*	79	1 (0.7)	79	N.A.	N.A.
***Heart***	**450**	**1 (0.7)**	**450**	**N.A.**	**N.A.**
*Journal of the American Geriatrics Society*	384	1 (0.7)	384	N.A.	N.A.
***Journal of Clinical Epidemiology***	**173**	**1 (0.7)**	**173**	**N.A.**	**N.A.**
*Journal of Clinical Psychopharmacology*	162	1 (0.7)	162	N.A.	N.A.
***Journal of Infectious Disease***	**760**	**1 (0.7)**	**760**	**N.A.**	**N.A.**
*Medical Care*	162	1 (0.7)	162	N.A.	N.A.
***Medical Journal of Australia***	**598**	**1 (0.7)**	**598**	**N.A.**	**N.A.**
*Rheumatology*	339	1 (0.7)	339	N.A.	N.A.
***Spine***	**604**	**1 (0.7)**	**604**	**N.A.**	**N.A.**
*Thorax*	336	1 (0.7)	336	N.A.	N.A.
*Total*		144		N.A.	N.A.
*Correlation with SCI Impact Factors-correlation coefficient (P-value)*	N.A.	0.546 (< 0.001)	N.A.	N.A.	N.A.

^†^*EBM *does not include a listing of important but not abstracted articles ("Other Articles Noted" section); *Articles abstracted are those that pass methodological criteria and are deemed to be the most important by a team of practicing general/family practitioners; **The number of articles needed to read (NNR) is a measure of the ratio of number of relevant articles (abstracted) divided into the total number of articles for each title; ***SCI Impact Factors not available for analysis; N.A. Not applicable. Data are for 2000. Note that the AHRQ/AHCPR reports were not considered journal titles and read as such for this report.

**Table 4:** *EBN *(general practice nursing) journal-specific content of high-quality, clinically relevant articles

**Journal title**	**Number articles in 2000 in journal**	**Number abstracted (% included in *EBN*)**	**NNR for abstracted article**	Number abstracted or listed (% included in *EBN*)*	NNR for abstracted or listed**
*Qualitative Health Research*	60	10 (10.4)	6.0	14 (4.6)	4.7
Cochrane Database of Systematic Review***	**444**	**8 (8.3)**	**55.5**	**33 (10.9)**	**13.5**
*Pediatrics*	811	8 (8.3)	101.4	19 (6.3)	42.7
***JAMA***	**1930**	**7 (7.3)**	**275.7**	**16 (5.3)**	**120.6**
*Lancet*	3858	6 (6.3)	643.0	12 (4.0)	321.5
***BMJ***	**3428**	**5 (5.2)**	**685.6**	**16 (5.3)**	**214.3**
*Journal of Advanced Nursing*	611	5 (5.2)	122.2	14 (4.6)	43.6
***American Journal of Medicine***	**434**	**3 (3.1)**	**144.7**	**6 (2.0)**	**72.3**
*Critical Care Medicine*	977	3 (3.1)	325.7	3 (1.0)	325.7
Health Psychology***	**79**	**3 (3.1)**	**26.3**	**5 (1.6)**	**15.8**
*Stroke*	609	3 (3.1)	203.0	5 (1.6)	121.8
***Archives of Internal Medicine***	**620**	**2 (2.1)**	**310**	**16 (5.3)**	**38.8**
*Archives of Pediatric and Adolescent Medicine*	273	2 (2.1)	136.5	5 (1.6)	57.4
***CMAJ (formerly Canadian Medical Association Journal)***	**1007**	**2 (2.1)**	**503.5**	**3 (1.0)**	**335.7**
Health Education and Behavior***	67	2 (2.1)	33.5	2 (0.7)	33.5
***Journal of Pediatrics***	**137**	**2 (2.1)**	**68.5**	**6 (2.0)**	**22.8**
*Social Science and Medicine*	302	2 (2.1)	151	5 (1.6)	60.4
***Age and Ageing***	**767**	**1 (1.0)**	**767**	**2 (0.7)**	**383.5**
*Annals of Internal Medicine*	602	1 (1.0)	602	6 (2.0)	100.3
***Annals of Surgery***	**301**	**1 (1.0)**	**301**	**1 (0.3)**	**301**
*ANS Advances in Nursing Sciences*	25	1 (1.0)	25	3 (1.0)	8.3
***Applied Nursing Research***	**40**	**1 (1.0)**	**40**	**2 (0.7)**	**20**
*Archives of Disease in Childhood Neonatal and Fetal Edition*	157	1 (1.0)	157	3 (1.0)	52.3
***Archives of General Psychiatry***	**161**	**1 (1.0)**	**161**	**4 (1.3)**	**40.3**
*Birth*	105	1 (1.0)	105	1 (0.3)	105
***British Journal of General Practice***	**453**	**1 (1.0)**	**453**	**4 (1.3)**	**113.3**
*British Journal of Surgery*	402	1 (1.0)	402	2 (0.7)	201
***Canadian Journal of Gastroenterology***	**145**	**1 (1.0)**	**145**	**1 (0.3)**	**145**
*Canadian Journal of Infection Control*	31	1 (1.0)	31	1 (0.3)	31
***Image Journal of Nursing Scholarship***	**94**	**1 (1.0)**	**94**	**2 (0.7)**	**47**
*Journal of the American Geriatric Society*	384	1 (1.0)	384	4 (1.3)	96
***Journal of Clinical Epidemiology***	**173**	**1 (1.0)**	**173**	**5 (1.6)**	**57.7**
*Journal of Clinical Nursing*	107	1 (1.0)	107	3 (1.0)	35.7
***Journal of Consulting and Clinical Psychology***	**122**	**1 (1.0)**	**122**	**5 (1.6)**	**24.4**
*Journal of Manipulative and Physical Therapy*	117	1 (1.0)	117	1 (0.3)	117
***Midwifery***	**68**	**1 (1.0)**	**68**	**3 (1.0)**	**22.7**
*New England Journal of Medicine*	1530	1 (1.0)	1530	4 (1.3)	382.5
***Pain***	**269**	**1 (1.0)**	**269**	**1 (0.3)**	**269**
*Psychosomatic Medicine*	106	1 (1.0)	106	1 (0.3)	106
***Western Journal of Nursing Research***	**99**	**1 (1.0)**	**99**	**1 (0.3)**	**99**
*Acta Psychiatrica Scandinavica*	255	0 (0)	Infinity	1 (0.3)	255
***Addiction***	**295**	**0 (0)**	**Infinity**	**1 (0.3)**	**295**
*American Journal of Cardiology*	850	0 (0)	Infinity	1 (0.3)	850
***American Journal of Epidemiology***	**362**	**0 (0)**	**Infinity**	**11 (3.6)**	**32.9**
*American Journal of Gastroenterology*	923	0 (0)	Infinity	1 (0.3)	923
***American Journal of Obstetrics and Gynecology***	**704**	**0 (0)**	**Infinity**	**2 (0.7)**	**352**
*American Journal of Public Health*	363	0 (0)	Infinity	4 (1.3)	90.8
***American Journal of Respiratory and Critical Care Medicine***	**783**	**0 (0)**	**Infinity**	**1 (0.3)**	**783**
*Archives of Diseases in Childhood*	392	0 (0)	Infinity	2 (0.7)	196
***British Journal of Psychiatry***	**335**	**0 (0)**	**Infinity**	**2 (0.7)**	**177.5**
*Canadian Journal of Nursing Research*	35	0 (0)	Infinity	1 (0.3)	35
***Canadian Journal of Psychiatry***	**179**	**0 (0)**	**Infinity**	**1 (0.3)**	**179**
*Cancer*	786	0 (0)	Infinity	1 (0.3)	786
***Cancer Nursing***	**61**	**0 (0)**	**Infinity**	**1 (0.3)**	**61**
*Chest*	882	0 (0)	Infinity	1 (0.3)	882
***Child Development***	**141**	**0 (0)**	**Infinity**	**1 (0.3)**	**141**
*Gut*	446	0 (0)	Infinity	1 (0.3)	446
***Heart and Lung***	**59**	**0 (0)**	**Infinity**	**1 (0.3)**	**59**
*International Journal of Geriatric Psychiatry*	169	0 (0)	Infinity	1 (0.3)	169
***Journal of American Academy of Child and Adolescent Psychiatry***	**300**	**0 (0)**	**Infinity**	**1 (0.3)**	**300**
*Journal of Child Psychology and Psychiatry*	99	0 (0)	Infinity	1 (0.3)	99
***Journal of Epidemiology and Community Health***	**205**	**0 (0)**	**Infinity**	**3 (1.0)**	**68.3**
*Journal of Family Practice*	263	0 (0)	Infinity	7 (2.3)	37.6
*Journal of General Internal Medicine*	155	0 (0)	Infinity	1 (0.3)	155
***Journal of Pediatric Nursing***	**68**	**0 (0)**	**Infinity**	**2 (0.7)**	**34**
*Journal of Pediatric and Oncology Nursing*	32	0 (0)	Infinity	1 (0.3)	32
***Medical Journal of Australia***	**598**	**0 (0)**	**Infinity**	**1 (0.3)**	**598**
*Nursing Research*	51	0 (0)	Infinity	3 (1.0)	17
***Obstetrics and Gynecology***	**478**	**0 (0)**	**Infinity**	**3 (1.0)**	**159.3**
*Patient Education and Counseling*	94	0 (0)	Infinity	3 (1.0)	31.3
***Schizophrenia Bulletin***	**80**	**0 (0)**	**Infinity**	**2 (0.7)**	**40**
*Spine*	604	0 (0)	Infinity	2 (0.7)	302
***Thorax***	**336**	**0 (0)**	**Infinity**	**1 (0.3)**	**336**
*Total*		96		305	
*Correlation with SCI Impact Factors-correlation coefficient (P-value)*	N.A.	0.096 (0.57)	N.A.	0.256 (0.038)	N.A.

*Articles abstracted are those that pass methodological criteria and are deemed to be the most important by practicing nurses. The other articles listed are articles with the same high-quality methods but are considered to be slightly less important clinically by practicing nurses; **The number of articles needed to read (NNR) is a measure of the ratio of number of relevant articles (abstracted or combined abstracted or listed) divided into the total number of articles for each journal title; ***SCI Impact Factor not available for analysis; N.A. Not applicable or available. Data are for 2000. Note that the AHRQ/AHCPR reports were not considered journal titles and read as such for this report.

**Table 5:** *EBMH *(mental health) journal-specific content of high-quality, clinically relevant articles

**Journal title**	**Number articles in 2000 in journal**	**Number abstracted (% included in *EBMH*)**	**NNR for abstracted article**	Number abstracted or listed (% included in *EBMH*)*	NNR for abstracted or listed**
*Archives of General Psychiatry*	161	12 (12.5)	20.1	14 (8.2)	11.5
Cochrane Database of Systematic Reviews***	**444**	**6 (6.3)**	**74.0**	**27 (15.9)**	**16.4**
*American Journal of Psychiatry*	508	5 (5.2)	101.6	9 (5.3)	56.4
***British Journal of Psychiatry***	**335**	**5 (5.2)**	**67.0**	**8 (4.7)**	**41.9**
*JAMA*	1930	5 (5.2)	386.0	9 (5.3)	214.4
***Lancet***	**3858**	**5 (5.2)**	**771.6**	**7 (4.1)**	**551.1**
*International Journal of Geriatric Psychiatry*	169	5 (5.2)	33.8	8 (4.7)	21.1
***Journal of the American Academy of Child and Adolescent Psychiatry***	**300**	**4 (4.2)**	**75.0**	**4 (2.4)**	**75.0**
Journal of Consulting and Clinical Psychology***	122	4 (4.2)	30.5	7 (4.1)	17.4
***BMJ***	**3428**	**3 (3.1)**	**1142.7**	**4 (2.4)**	**857.0**
*Journal of Clinical Psychopharmacology*	162	3 (3.1)	54.0	3 (1.7)	54.0
***Schizophrenia Bulletin***	**80**	**3 (3.1)**	**26.7**	**4 (2.4)**	**20.0**
*Annals of Internal Medicine*	602	2 (2.1)	301.5	2 (1.2)	301.5
Journal of Child Psychology and Psychiatry, and Allied Disciplines***	**99**	**2 (2.1)**	**49.5**	**3 (1.7)**	**33.0**
*Archives of Internal Medicine*	620	2 (2.1)	310	3 (1.7)	206.7
***Journal of Clinical Epidemiology***	**173**	**2 (2.1)**	**86.5**	**2 (1.2)**	**86.5**
*Journal of Family Practice*	263	2 (2.1)	131.5	2 (1.2)	131.5
***New England Journal of Medicine***	**1530**	**2 (2.1)**	**765.0**	**2 (1.2)**	**765.0**
*Psychosomatic Medicine*	106	2 (2.1)	53.0	3 (1.7)	35.3
***Acta Psychiatric Scandinavica***	**255**	**1 (1.0)**	**255**	**2 (1.2)**	**127.5**
*Addiction*	295	1 (1.0)	295	4 (2.4)	73.8
***Age and Ageing***	**181**	**1 (1.0)**	**181**	**1 (0.6)**	**181**
*American Journal of Public Health*	363	1 (1.0)	363	1 (0.6)	363
***Archives of Family Medicine (no longer published)***	**230**	**1 (1.0)**	**230**	**1 (0.6)**	**230**
*Behaviour Therapy*	44	1 (1.0)	44	3 (1.7)	14.7
***British Journal of General Practice***	**453**	**1 (1.0)**	**453**	**1 (0.6)**	**453**
*British Journal of Geriatric Psychiatry*	N.A.	1 (1.0)	N.A.	1 (0.6)	N.A.
***Canadian Journal of Psychiatry***	**179**	**1 (1.0)**	**179**	**1 (0.6)**	**179**
*Child Development*	141	1 (1.0)	141	2 (1.2)	70.5
***CMAJ (formerly Canadian Medical Association Journal)***	**1007**	**1 (1.0)**	**1007**	**1 (0.6)**	**1007**
*Image Journal of Nursing Scholarship*	94	1 (1.0)	94	1 (0.6)	94
***Journal of Advanced Nursing***	**611**	**1 (1.0)**	**611**	**1 (0.6)**	**611**
*Journal of Affective Disorders*	154	1 (1.0)	154	1 (0.6)	154
***Journal of Neurology Neurosurgery and Psychiatry***	**83**	**1 (1.0)**	**83**	**2 (1.2)**	**41.5**
*Medical Care*	162	1 (1.0)	162	1 (0.6)	162
***Pain***	**269**	**1 (1.0)**	**269**	**1 (0.6)**	**269**
*Pediatrics*	811	1 (1.0)	811	2 (1.2)	405.5
***Psychiatric Services***	**356**	**1 (1.0)**	**356**	**3 (1.7)**	**118.7**
*Psychology and Aging*	55	1 (1.0)	55	1 (0.6)	55
***Research in Nursing Health***	**55**	**1 (1.0)**	**55**	**1 (0.6)**	**55**
*Social Science and Medicine*	302	1 (1.0)	302	1 (0.6)	302
***American Journal of Medicine***	**435**	**0 (0)**	**Infinity**	**2 (1.2)**	**217.5**
AHCOR/AHRQ Reports***	N.A.	0 (0)	Infinity	3 (1.7)	N.A.
***Archives of Physical Medicine and Rehabilitation***	**337**	**0 (0)**	**Infinity**	**1 (0.6)**	**337**
*Australian and New Zealand Journal of Psychiatry*	214	0 (0)	Infinity	1 (0.6)	214
***Journal of Clinical Nursing***	**107**	**0 (0)**	**Infinity**	**1 (0.6)**	**107**
*Journal of Psychosomatic Research*	118	0 (0)	Infinity	2 (1.2)	59
***Neurology***	**1334**	**0 (0)**	**Infinity**	**1 (0.6)**	**1334**
*Psychosomatic Medicine*	106	0 (0)	Infinity	1 (0.6)	106
*Total*		96		170	
*Correlation with SCI Impact Factors-correlation coefficient (P-value)*	N.A.	0.386 (0.02)	N.A.	0.381 (0.01)	N.A.

*Articles abstracted are those that pass methodological criteria and are deemed to be the most important by practicing mental health professionals. The other articles listed are articles with the same high-quality methods but are considered to be slightly less important clinically by mental health professionals; **The number of articles needed to read (NNR) is a measure of the ratio of number of relevant articles (abstracted or combined abstracted or B-listed) divided into the total number of articles for each journal title; ***SCI Impact Factor not available for analysis; N.A. Not applicable or not available. Data are for 2000. Note that the AHRQ/AHCPR reports were not considered journal titles and read as such for this report.
