# Supplementary material for: What do evidence-based secondary journals tell us about the publication of clinically important articles in primary healthcare journals?
Source: BMC Med. 2004 Sep 6;2:33. doi: 10.1186/1741-7015-2-33 (PMC518974; doi:10.1186/1741-7015-2-33)
Supplement: Additional File 1 — includes a list of the 170 journals read for 2000 along with the number of articles reviewed, the number and percentage that passed criteria, and the NNR (number of articles that are needed to be read to obtain one that is clinically relevant and has high-quality methods). The file name is "Publishing Important Articles Appendix.doc" and it is in Word 2000 format. [file 1741-7015-2-33-S1.doc]

# Appendix

Journals read for 2000 with total number of articles rated and the
number of original and review articles that pass criteria

| Journal title | **Articles assessed** | **Articles that passed criteria** | **Percent pass** | **NNR*** |
| --- | --- | --- | --- | --- |
| *Cochrane Database of Systematic Reviews* | 444 | 422 | 95 | 2 |
| ***Lancet*** | **3858** | **134** | **3.5** | **29** |
| *Journal of Clinical Oncology* | 650 | 100 | 15.4 | 7 |
| ***BMJ*** | **3428** | **93** | **2.7** | **38** |
| *Circulation* | 1351 | 92 | 6.8 | 15 |
| ***Journal of Advanced Nursing*** | **611** | **92** | **15.1** | **7** |
| *Obstetrics & Gynecology* | 478 | 88 | 18.4 | 6 |
| ***JAMA*** | **1930** | **87** | **4.5** | **23** |
| New England Journal of Medicine | 1530 | 83 | 5.4 | 19 |
| ***Archives of Internal Medicine*** | **620** | **81** | **13.1** | **8** |
| *Journal of the American College of Cardiology* | 707 | 76 | 10.7 | 10 |
| ***Pediatrics*** | **811** | **76** | **9.4** | **11** |
| *American Journal of Cardiology* | 850 | 72 | 8.5 | 12 |
| ***American Journal of Obstetrics & Gynecology*** | **704** | **72** | **10.2** | **10** |
| *Critical Care Medicine* | 977 | 70 | 7.2 | 14 |
| ***Chest*** | **882** | **66** | **7.5** | **14** |
| *Stroke* | 609 | 59 | 9.7 | 11 |
| ***Neurology*** | **1334** | **58** | **4.3** | **24** |
| *American Journal of Gastroenterology* | 923 | 56 | 6.1 | 17 |
| ***Diabetes Care*** | **529** | **54** | **10.2** | **10** |
| Annals of Internal Medicine | 602 | 52 | 8.6 | 11 |
| ***American Journal of Respiratory & Critical Care Medicine*** | **783** | **43** | **5.5** | **19** |
| *BJOG (formerly British Journal of Obstetrics and Gynaecology)* | 334 | 43 | 12.9 | 8 |
| ***Fertility & Sterility*** | **563** | **42** | **7.5** | **14** |
| *Journal of Infectious Diseases* | 760 | 40 | 5.3 | 19 |
| ***Social Science & Medicine*** | **302** | **40** | **13.2** | **8** |
| Journal of Pediatrics | 444 | 39 | 8.8 | 12 |
| ***Cancer*** | **786** | **38** | **4.8** | **21** |
| *Journal of Rheumatology* | 657 | 37 | 5.6 | 18 |
| ***Journal of the American Geriatrics Society*** | **384** | **35** | **9.1** | **11** |
| *American Journal of Medicine* | 435 | 32 | 7.4 | 14 |
| ***Qualitative Health Research*** | **60** | **29** | **48.3** | **3** |
| *Spine* | 604 | 28 | 4.6 | 22 |
| ***American Journal of Epidemiology*** | **362** | **27** | **7.5** | **14** |
| *Annals of Emergency Medicine* | 294 | 27 | 9.2 | 11 |
| ***British Journal of Psychiatry*** | **335** | **27** | **8.1** | **13** |
| *Radiology* | 654 | 27 | 4.1 | 25 |
| ***Journal of Family Practice*** | **263** | **26** | **9.9** | **11** |
| *Journal of Clinical Nursing* | 107 | 25 | 23.4 | 5 |
| ***British Journal of Surgery*** | **402** | **24** | **6** | **17** |
| *Journal of Clinical Psychopharmacology* | 162 | 24 | 14.8 | 7 |
| ***Thorax*** | **336** | **24** | **7.1** | **15** |
| *Annals of Surgery* | 301 | 23 | 7.6 | 14 |
| ***Archives of Physical Medicine & Rehabilitation*** | **337** | **23** | **6.8** | **15** |
| *Journal of Consulting & Clinical Psychology* | 122 | 23 | 18.9 | 6 |
| ***American Journal of Psychiatry*** | **508** | **22** | **4.3** | **24** |
| *Archives of General Psychiatry* | 161 | 22 | 13.7 | 8 |
| ***British Journal of General Practice*** | **453** | **22** | **4.9** | **21** |
| *Archives of Pediatrics & Adolescent Medicine* | 273 | 21 | 7.7 | 13 |
| ***Archives of Disease in Childhood*** | **392** | **19** | **4.8** | **21** |
| *Heart* | 450 | 19 | 4.2 | 24 |
| ***Journal of Clinical Psychiatry*** | **335** | **19** | **5.7** | **18** |
| *Journal of General Internal Medicine* | 155 | 19 | 12.3 | 9 |
| ***AJR American Journal of Roentgenology*** | **767** | **18** | **2.3** | **44** |
| *Archives of Disease in Childhood Fetal & Neonatal Edition* | 157 | 18 | 11.5 | 9 |
| ***International Journal of Geriatric Psychiatry*** | **169** | **18** | **10.7** | **10** |
| *American Journal of Surgery* | 310 | 17 | 5.5 | 19 |
| ***Archives of Family Medicine (no longer published)*** | **230** | **17** | **7.4** | **14** |
| *Cancer Nursing* | 61 | 17 | 27.9 | 4 |
| ***CMAJ (formerly Canadian Medical Association Journal)*** | **1007** | **17** | **1.7** | **59** |
| Diabetic Medicine | 188 | 17 | 9 | 12 |
| ***Pain*** | **269** | **17** | **6.3** | **16** |
| *Western Journal of Nursing Research* | 99 | 17 | 17.2 | 6 |
| ***Journal of Affective Disorders*** | **154** | **16** | **10.4** | **10** |
| *Journal of Epidemiology & Community Health* | 205 | 16 | 7.8 | 13 |
| ***Arthritis & Rheumatism*** | **440** | **15** | **3.4** | **30** |
| *Gut* | 446 | 15 | 3.4 | 30 |
| ***Journal of Neurology Neurosurgery & Psychiatry*** | **478** | **15** | **3.1** | **33** |
| *Journal of Trauma Injury Infection & Critical Care* | 562 | 14 | 2.5 | 40 |
| ***Journal of Vascular Surgery*** | **544** | **14** | **2.6** | **39** |
| *Research in Nursing & Health* | 55 | 14 | 25.5 | 4 |
| ***Clinical Orthopaedics & Related Research*** | **472** | **13** | **2.8** | **36** |
| *Journal of Psychosomatic Research* | 118 | 13 | 11 | 10 |
| ***Psychological Medicine*** | **142** | **13** | **9.2** | **11** |
| *Addiction* | 295 | 12 | 4.1 | 25 |
| ***Gastroenterology*** | **543** | **12** | **2.2** | **46** |
| *Journal of Internal Medicine* | 177 | 12 | 6.8 | 15 |
| ***Patient Education & Counseling*** | **94** | **12** | **12.8** | **8** |
| *Rheumatology* | 339 | 12 | 3.5 | 29 |
| ***Annals of the Rheumatic Diseases*** | **266** | **11** | **4.1** | **25** |
| *Archives of Surgery* | 330 | 11 | 3.3 | 31 |
| ***Family Practice*** | **117** | **11** | **9.4** | **11** |
| Health Psychology | 79 | 11 | 13.9 | 8 |
| ***Heart & Lung*** | **59** | **11** | **18.6** | **6** |
| *Medical Care* | 162 | 11 | 6.8 | 15 |
| ***Midwifery*** | **68** | **11** | **16.2** | **7** |
| *Psychiatric Services* | 356 | 11 | 3.1 | 33 |
| ***Public Health Nursing*** | **62** | **11** | **17.7** | **6** |
| *Acta Orthopaedica Scandinavica* | 121 | 10 | 8.3 | 12 |
| ***American Journal of Public Health*** | **363** | **10** | **2.8** | **36** |
| *Canadian Journal of Public Health Revue Canadienne de Sante Publique* | 127 | 10 | 7.9 | 13 |
| ***Hypertension*** | **419** | **10** | **2.4** | **42** |
| *Journal of Nursing Scholarship* | 94 | 10 | 10.6 | 10 |
| ***Age & Ageing*** | **181** | **9** | **5** | **20** |
| *Australian & New Zealand Journal of Psychiatry* | 214 | 9 | 4.2 | 24 |
| ***Journal of Clinical Epidemiology*** | **173** | **9** | **5.2** | **20** |
| *Journal of the American Academy of Child & Adolescent Psychiatry* | 300 | 9 | 3 | 34 |
| ***Journal of Arthroplasty*** | **171** | **8** | **4.7** | **22** |
| *Medical Journal of Australia* | 598 | 8 | 1.3 | 77 |
| ***ANS Advances in Nursing Science*** | **25** | **7** | **28** | **4** |
| *Arthroscopy* | 152 | 7 | 4.6 | 22 |
| ***Clinical Pediatrics*** | **152** | **7** | **4.6** | **22** |
| Health Education & Behavior | 67 | 7 | 10.4 | 10 |
| ***Journal of Bone & Joint Surgery American Volume*** | **360** | **7** | **1.9** | **53** |
| *Journal of Bone & Joint Surgery British Volume* | 315 | 7 | 2.2 | 46 |
| ***Journal of Hand Surgery British Volume*** | **161** | **7** | **4.3** | **24** |
| *Journal of the American College of Surgeons* | 233 | 7 | 3 | 34 |
| ***Schizophrenia Bulletin*** | **80** | **7** | **8.8** | **12** |
| *Surgery* | 368 | 7 | 1.9 | 53 |
| ***Western Journal of Medicine*** | **445** | **7** | **1.6** | **63** |
| *Acta Psychiatrica Scandinavica* | 255 | 6 | 2.4 | 42 |
| ***Birth*** | **105** | **6** | **5.7** | **18** |
| *Canadian Journal of Nursing Research* | 35 | 6 | 17.1 | 6 |
| ***Clinical Nursing Research*** | **32** | **6** | **18.8** | **6** |
| *Journal of Child Psychology & Psychiatry & Allied Disciplines* | 99 | 6 | 6.1 | 17 |
| ***Journal of Neurosurgery*** | **549** | **6** | **1.1** | **91** |
| *Journal of Orthopaedic Trauma* | 128 | 6 | 4.7 | 22 |
| ***Journal of Pediatric Nursing*** | **68** | **6** | **8.8** | **12** |
| *Nursing Research* | 51 | 6 | 11.8 | 9 |
| ***Plastic & Reconstructive Surgery*** | **940** | **6** | **0.6** | **167** |
| *Behaviour Therapy* | 44 | 5 | 11.4 | 9 |
| ***Canadian Journal of Psychiatry Revue Canadienne de Psychiatrie*** | **179** | **5** | **2.8** | **36** |
| Journal of Manipulative & Physiological Therapeutics | 117 | 5 | 4.3 | 24 |
| ***Neonatal Network*** | **108** | **5** | **4.6** | **22** |
| *American Journal of Sports Medicine* | 179 | 4 | 2.2 | 46 |
| ***Archives of Neurology*** | **313** | **4** | **1.3** | **77** |
| *Behaviour Research & Therapy* | 87 | 4 | 4.6 | 2 |
| ***Canadian Respiratory Journal*** | **68** | **4** | **5.9** | **17** |
| *Child Development* | 141 | 4 | 2.8 | 36 |
| ***Journal of Abnormal Psychology*** | **93** | **4** | **4.3** | **24** |
| *Journal of Child & Adolescent Psychopharmacology* | 42 | 4 | 9.5 | 11 |
| ***Psychosomatic Medicine*** | **106** | **4** | **3.8** | **27** |
| *Annals of Medicine* | 77 | 3 | 3.9 | 26 |
| ***Applied Nursing Research*** | **40** | **3** | **7.5** | **14** |
| *Archives of Medical Research* | 227 | 3 | 1.3 | 77 |
| ***Canadian Journal of Cardiology*** | **207** | **3** | **1.4** | **72** |
| *Canadian Journal of Gastroenterology* | 145 | 3 | 2.1 | 48 |
| ***Canadian Journal of Surgery*** | **115** | **3** | **2.6** | **39** |
| *Injury* | 212 | 3 | 1.4 | 72 |
| ***Journal of Clinical Child Psychology*** | **49** | **3** | **6.1** | **17** |
| *Journal of Pediatric Oncology Nursing* | 32 | 3 | 9.4 | 11 |
| ***Quality in Health Care*** | **49** | **3** | **6.1** | **17** |
| *Clinical & Investigative Medicine Medecine Clinique et Experimentale* | 76 | 2 | 2.6 | 39 |
| ***Journal of Abnormal Child Psychology*** | **46** | **2** | **4.3** | **24** |
| Journal of Clinical & Experimental Neuropsychology | 75 | 2 | 2.7 | 37 |
| ***Journal of Hand Surgery American Volume*** | **171** | **2** | **1.2** | **84** |
| *Journal of Neuropsychiatry & Clinical Neurosciences* | 83 | 2 | 2.4 | 42 |
| ***Journal of Pediatric Orthopedics*** | **168** | **2** | **1.2** | **84** |
| *Journal of the American Board of Family Practice* | 121 | 2 | 1.7 | 59 |
| ***Neurosurgery*** | **1638** | **2** | **0.1** | **1000** |
| *Psychological Assessment* | 52 | 2 | 3.8 | 27 |
| ***Psychology & Aging*** | **55** | **2** | **3.6** | **28** |
| *British Journal of Clinical Psychology* | 36 | 1 | 2.8 | 36 |
| ***Canadian Journal of Infection Control*** | **31** | **1** | **3.2** | **32** |
| *Canadian Journal of Infectious Diseases* | 57 | 1 | 1.8 | 56 |
| ***Development & Psychopathology*** | **43** | **1** | **2.3** | **44** |
| *General Hospital Psychiatry* | 63 | 1 | 1.6 | 63 |
| ***Journal of Autism & Developmental Disorders*** | **90** | **1** | **1.1** | **91** |
| *Journal of Cutaneous Medicine & Surgery* | 54 | 1 | 1.9 | 53 |
| ***Journal of Neurosurgery Spine*** | **88** | **1** | **1.1** | **91** |
| *Journal of the American Medical Informatics Association* | 72 | 1 | 1.4 | 72 |
| ***Paediatrics & Child Health*** | **81** | **1** | **1.2** | **84** |
| *Pediatric Radiology* | 224 | 1 | 0.4 | 250 |
| ***Clinical Psychology Review*** | **49** | **0** | **0** | **Infinity** |
| *Family Planning Perspectives* | 143 | 0 | 0 | Infinity |
| ***Foot and Ankle International*** | **169** | **0** | **0** | **Infinity** |
| *Journal of Orthopaedic Research* | 139 | 0 | 0 | Infinity |
| ***Psychiatry Interpersonal and Biological Processes*** | **42** | **0** | **0** | **Infinity** |
| *Psychological Bulletin* | 49 | 0 | 0 | Infinity |

*NNR is the number of articles needed to be read to provide one that has high-quality methods and is clinically important. Articles have not been screened for direct clinical relevance beyond basic criteria of having at least one clinically important outcome.
